# Correlating the Segmental Relaxation Time of Polystyrene †

**DOI:** 10.3390/polym16152154

**Published:** 2024-07-29

**Authors:** C. A. Hieber

**Affiliations:** Sibley School of Mechanical and Aerospace Engineering, Cornell University, Ithaca, NY 14853, USA; cah31@cornell.edu

**Keywords:** equilibrium relaxation time, glass–rubber transition, glass transition temperature, polystyrene, segmental relaxation time, VFTH model

## Abstract

A previous related paper dealing with the density relaxation of polystyrene (PS) has shown that the equilibrium relaxation time (τ_eq_) has a purely exponential temperature dependence (ETD) below ≈100 °C. Such an ETD is now also confirmed based upon available dielectric spectra data for PS. By combining the ETD behavior of τ_eq_ (or a_T_) at low temperatures with a VFTH behavior at higher temperatures (based mainly on available recoverable shear compliance data), a composite correlation for τ_eq_ (or a_T_) is developed, which is continuous with continuous slope at a crossover temperature that is found to be 99.22 °C, where τ_eq_ = 92.15 s. This composite representation is shown to describe (without any adjustable parameters) available independent data for the segmental relaxation time over a finite range both above and below T_crossover_ (i.e., the glass transition temperature).

## 1. Introduction

In a recent paper by Hieber [1], based upon the density relaxation of polystyrene at atmospheric pressure, it was shown that the equilibrium relaxation time is characterized by a purely exponential temperature dependence over the experimental range available in the literature, reaching (under equilibrium) about 16 °C below the nominal glass transition temperature. Such results were shown (in the same paper) to be compatible with the stress relaxation data for polycarbonate of O’Connell and McKenna [2] as well as with the equilibrium dielectric compliance data for PVAc of Zhao and McKenna [3]; in both of these cases, the equilibrium state could again be reached at about 16 or 17 °C below the nominal glass transition temperature. {It is noted that the “T_g,nominal_” for PS is typically considered to be 373 °K (i.e., essentially 100 °C), as has been done, for example, by Roland and Casalini [4] and He et al. [5]}.

In the present paper, it will be shown for polystyrene that the equilibrium relaxation time τ_eq_(T) from Hieber [1] can be extended to temperatures above the nominal glass transition temperature by making use of the temperature shift factor ($a_{T}$) in the glass–rubber transition obtained from available independent data (in terms of recoverable shear creep compliance as well as stress relaxation) from the literature. It will be shown that this composite representation of τ_eq_(T) describes (without any adjustable parameters) the available data for the segmental correlation time of PS over a temperature range extending both above and below the (nominal) glass transition temperature.

## 2. Extending τ_eq_(T) above T_g_

Based on the results from fitting the cumulative density relaxation data for PS from Hieber [1], the resulting equilibrium relaxation time (at atmospheric pressure) is given by

(1) $$\tau_{\mathrm{eq}}\left(T\right)=\mathrm{AA}\;\exp\left\{-\alpha_{3}\left(T\;-100\;{}^{\circ}C\right)\right\}$$

where

(2) $$\mathrm{AA}=49.18\;s,\;\alpha_{3}=0.805/{}^{\circ}C$$

**Figure 1:**
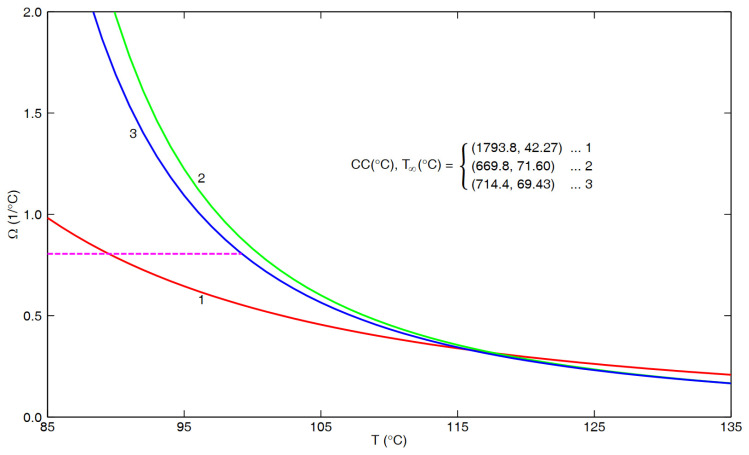
Results for Ω versus T. Curves based upon VFTH fits for flow regime (curve 1, from Appendix A) or glass–rubber transition (curves 2 and 3, from Appendix B). Dashed line based upon Equations (1) and (2).

Combining this with the results from Appendix A and Appendix B below, we arrive at the plot in Figure 1 in which the ordinate is a measure of the temperature sensitivity in terms of τ_eq_(T) or a_T_, namely,

(3) $$\Omega \;\equiv -\frac{d\ln\tau_{\mathrm{eq}}\;\left(T\right)}{\mathrm{dT}}$$

or

(4) $$\Omega \;\equiv -\frac{d\ln a_{T}}{\mathrm{dT}}$$

In particular, based upon the density relaxation data for PS from Hieber [1], we have that

(5) Ω=0.805/°C

over the interval between 83.87 °C and 100 °C.

On the other hand, the three curves in Figure 1 are all based upon the VFTH model [6,7,8], namely,

(6) $$a_{T}=\mathrm{BB}\;\exp\left(\frac{\mathrm{CC}}{\left(T-T_{\infty }\right)}\right)$$

such that

(7) $$\Omega =\frac{\mathrm{CC}}{{\left(T-T_{\infty }\right)}^{2}}$$

In particular, curve 1 in Figure 1 is based upon cumulative data from five sources [9,10,11,12,13] for the “flow regime” reported in Appendix A, with the measured temperatures ranging between 104.5 °C and 290 °C, and (CC, T_∞_) = (1793.8 °C, 42.27 °C), as given in Equation (A2). On the other hand, curves 2 and 3 are based upon results for the “glass–rubber transition” from Appendix B involving cumulative data from six sources [9,14,15,16,17,18] in the temperature range from 100 °C to 135 °C, with curve 2 corresponding to (CC, T_∞_) = (669.8 °C, 71.60 °C) from Equation (A4) of Appendix B, and curve 3 to (CC, T_∞_) = (714.4 °C, 69.43 °C) from Equation (A5).

**Figure A1:**
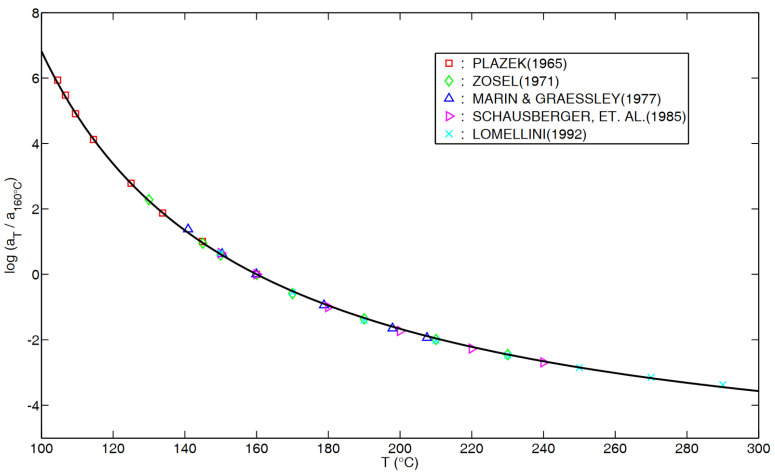
Cumulative data for the temperature sensitivity of $a_{T}$ for the flow regime of PS. Solid curve corresponds to VFTH model, Equations (A1) and (A2).

**Figure A2:**
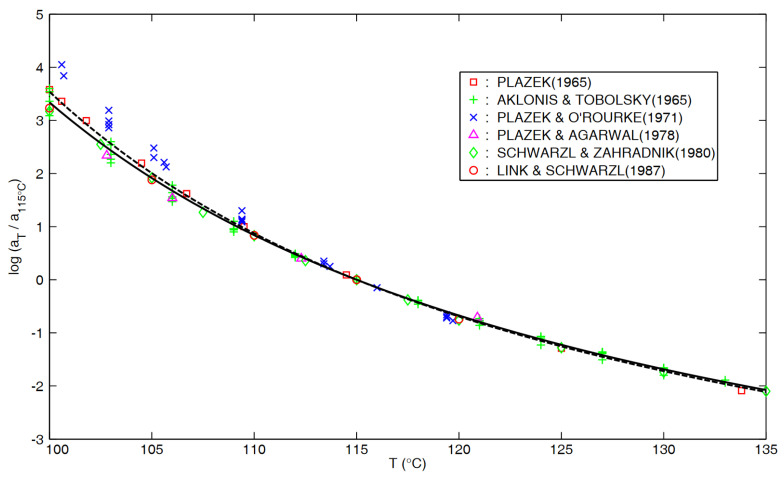
Cumulative data for the temperature sensitivity of a_T_ for the glass–rubber transition of PS. Both curves are based upon the VFTH model, with the dashed line corresponding to Equation (A4), and the solid line to Equation (A5).

Clearly, all three curves in Figure 1 were extended to temperatures below that of the underlying data (as presented in Figure A1 and Figure A2. Furthermore, it is expected that the present density relaxation results should be directly related to the “glass–rubber transition” results, both reflecting the local molecular behavior, whereas the “flow regime” results reflect long-range molecular motion. In addition, as documented in Appendix B, there is a basis for judging that curve 3 is more representative than curve 2. Accordingly, of special interest in Figure 1 is the intercept of curve 3 with the dashed result given by Equation (5), which occurs at T = 99.22 °C.

These results seem to strongly indicate that the VFTH behavior of the “glass–rubber transition” given by curve 3 in Figure 1 becomes replaced by the constant value given by Equation (5) at temperatures below the intersection point at 99.22 °C. In turn, this indicates that the singularity (at T ≡ T_∞_) in the VFTH equation is only an apparent singularity.

Making use of the results in Figure 1, it seems appropriate to introduce the term “T_crossover_” to denote where the dashed line and curve 3 intersect. Accordingly,

T_crossover_ = 99.22 °C (8)

where τ_eq_(T) is based on Equations (1) and (2) for T ≤ T_crossover_ and is extended above T_crossover_ by making use of curve 3 from Figure 1. That is, the resulting composite representation for τ_eq_ (T) is then given by

τ_eq_(T) = 49.18 s exp{ −(0.805/°C)(T − 100 °C)} (9)

for T ≤ T_crossover_, such that, from Equations (8) and (9),

τ_eq_(T_crossover_) = 92.15 s (10)

whereas, based upon curve 3 in Figure 1, for T > T_crossover_ we have that

(11) $$\tau_{\mathrm{eq}}(T)=92.15\;s\;\exp\;\left\{\frac{714.4\;℃}{T\;-69.43\;℃}-\frac{714.4\;℃}{99.22\;℃-69.43\;℃}\right\}$$

It is noted that the composite representation for τ_eq_(T), given by Equations (9) and (11), is continuous with a continuous slope at T_crossover_ {which follows from the definition of Ω in Equation (3)}. Furthermore, the value of T_crossover_ in Equation (8) is close to the “nominal T_g_” of PS, namely, 100° C. Accordingly, the result in Equation (10) is compatible with a convention typically associated with Angell [19], namely, that T_g,nominal_ is where τ_eq_ is on the order of 10^2^ s.

## 3. Comparison with Experimental Results for the Segmental Relaxation Time

**Figure 2:**
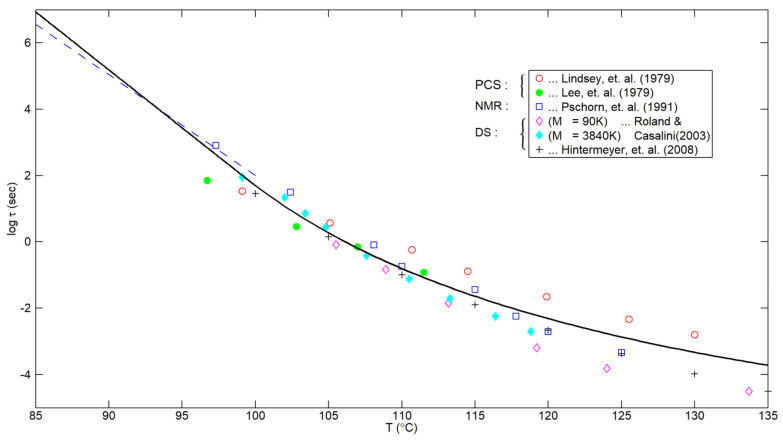
Predicted curve for τ_eq_(T) based upon Equations (8), (9) and (11) compared with PS data based on photon correlation spectroscopy [20,21], NMR [22], and dielectric spectroscopy [4,23]. The dashed line will be described in Section 6.

A resulting plot for τ_eq_(T) based upon Equations (9) and (11) is shown in Figure 2, together with corresponding data for PS based upon various experimental techniques. Despite the evident scatter, a definite correlation between the data and the composite curve (with no adjustable parameter) seems apparent.

It is worth stressing that the actual level of the τ_eq_(T) curve in Figure 2 is based upon the density relaxation results obtained in the earlier paper by Hieber [1]. On the other hand, the extension of the curve to higher temperatures (i.e., above T_crossover_) is based upon fitting cumulative results for a_T_ in the glass–rubber transition region, as presented in Appendix B of the current paper.

In observing Figure 2, it is noted that the experimental results from Roland and Casalini [4] are for two PS samples of significantly different M_w_, differing by a factor of 43, but the corresponding results for τ_eq_ differ by no more than a half decade. For comparison, if we were dealing with viscous flow, the characteristic time would be proportional to η_0_, which, for these large values of M_w_, would be proportional to M_w_ raised to the 3.4 power. Accordingly, the respective values of the viscous flow characteristic time for these two polymers would differ by a factor of 43 raised to the 3.4 power, i.e., 3.6 × 10^5^. Clearly, on such a scale, the present results in Figure 2 for the two PS samples are essentially coincident. Stated differently, these results indicate the dramatic difference in behavior of the current results in Figure 2, relating to the local segmental motion of PS, in contrast to the global molecular motion associated with viscous flow.

As a still further confirmation that the results in Figure 2 are independent of the molecular weight (if sufficiently large), the results for PS in Figure 2b of Hintermeyer et al. [23] are striking; they show that the curves for “lg τ_α_ (s) versus T (°K)” are essentially coincident for the three samples with the highest molecular weight (all of NMWD), namely, 96 K, 243 K, and 546 K. In fact, the corresponding data points from Hintermeyer et al. [23] shown in the current Figure 2 were taken from the right-most solid curve in their Figure 2b, which is representative of the high-Mw asymptote. It is noted that Hintermeyer et al. [23] determined “Tg” for each of their polymers as the temperature at which τα equals 100 s. From their Table 2, the corresponding values for the above three samples with high molecular weight were 372.6 °K (99.45 °C), 373.3 °K (100.15 °C), and 372.0 °K (98.85 °C), respectively.

## 4. Behavior at Higher Temperatures

**Figure 3:**
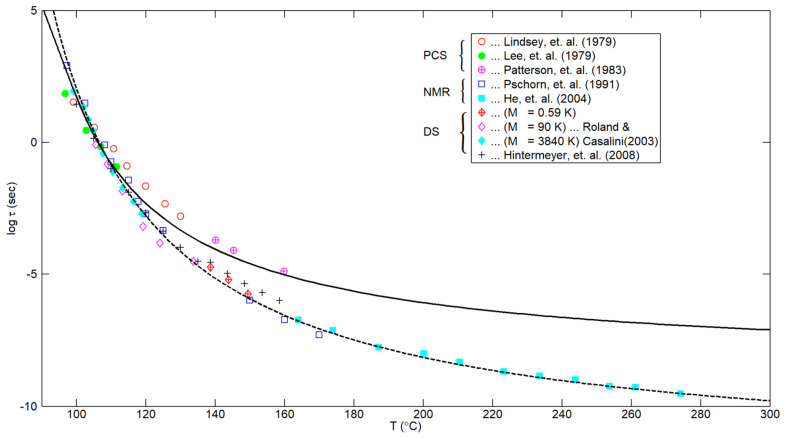
Predictions for the relaxation time τ(T) based on Equations (8), (9) and (11)—solid curve—or upon Equation (12)—dashed curve. Data based on photon correlation spectroscopy, NMR, and dielectric spectroscopy.

Whereas Figure 2 extends to only 135 °C (reflecting the underlying related data from Appendix B), Figure 3 extends the plot to higher temperatures. In particular, the solid curve is based upon Equations (9) and (11), as in Figure 2, whereas the dashed curve is based upon the empirical fit obtained by He et al. [5], namely,

(12) $$\tau_{\mathrm{seg},c}\left(T\right)=0.87\times 10^{-12}s\exp\left(\frac{1248\;{}^{\circ}C}{T-61.55{}^{\circ}C}\right)$$

for the segmental correlation time.

As noted in Figure 9 of He et al. [5], their data, based upon three PS samples of low M_w_ (namely, 2.05 K, 2.31 K, and 11.45 K), were “horizontally shifted by ΔT_g_, taking T_g_ = 373 °K for high molecular weight PS”. In particular, the highest-temperature data point from He et al. [5], as shown at 274 °C in the current Figure 3, corresponds to M_w_ = 2.05 K and ΔT_g_ = 54 °K/°C. Similarly, the three data points for M_w_ = 0.59 K from Roland and Casalini [4] were taken from their Figure 4 with a ΔTg of 119 °C. Furthermore, the five right-most data points from Hintermeyer et al. [23] shown in Figure 3 correspond to M_w_ = 1.350 K, as presented in their Figure 11, with a ΔT_g_ of 59 °C.

Evidently, with the exception of the data from Patterson et al. [24], the dashed curve clearly describes the high-temperature data in Figure 3 quite well. (It should be noted that the data from Lindsey et al. [20] and Patterson et al. [24] are from the same laboratory.) As indicated in Figure 9 of He et al. [5], their higher-temperature NMR data merge well with the lower-temperature NMR data of Pschorn et al. [22]. Furthermore (as seen in Figure 3), the correlation given by Equation (12) seems to be substantiated by the DS measurements obtained independently by Roland and Cassalini [4] and by Hintermeyer et al. [23].

It should be noted that Equation (12) gives a value of about 10^2^ s at 100 °C (often taken as the nominal glass transition temperature for PS). This is consistent with a convention typically associated with Angell [19], which was explicitly employed by Roland and Casalini [4] and by Hintermeyer et al. [23].

In closing this section, one might also consider the limiting behavior of the relaxation time at a hypothetically high temperature (i.e., as T → ∞), namely, “τ_∞_”. In particular, from Equations (11) and (12) above, we obtain the respective values of 3.546 × 10^−9^ s and 0.87 × 10^−12^ s. On the other hand, Boyd and Smith [25] noted that the limiting behavior (as T→ ∞) of various modes seem to converge on a time scale of picoseconds (10^−12^ s), corresponding to intramolecular and torsional oscillations. Hence, it would seem that Equation (12) would be more appropriate than Equation (11). But this is complicated by the generally accepted idea [26,27] that the WLF (or VFTH) model should be replaced by an Arrhenius behavior at sufficiently high temperatures. If that is performed in the case of Equation (11), supposing that the VFTH model in Equation (11) is replaced by an Arrhenius behavior at T = T*, with their values and first derivatives being continuous, it can be verified that $\tau_{\infty }\;≅\;10^{-13}\;s$ if $T^{*}≅\;222\;{}^{\circ}C$ (495 °K), and 10^−12^ s if $T^{*}≅\;242\;{}^{\circ}C$ (515 °K). That is, these values indicate that the Arrhenius behavior would decay more rapidly than the VFTH model at these higher temperatures and that the resulting values for τ_∞_ based upon such a composite VFTH/Arrhenius model would not be unreasonable.

## 5. Further Considerations

**Figure 4:**
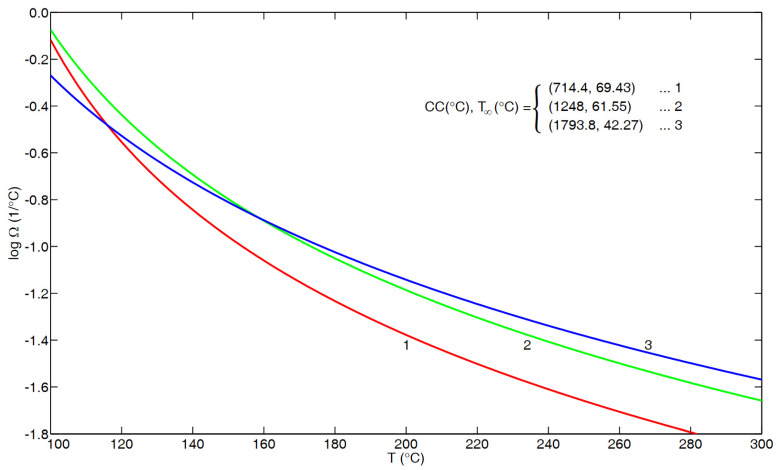
Results for Ω versus T based upon three VFTH fits, namely, Equation (A5) from Appendix B (“glass–rubber transition”), curve 1; Equation (12) from He et al. [5], curve 2; Equation (A2) from Appendix A (“flow regime”), curve 3.

Figure 4 compares the results based upon the VFTH fits in Equations (12), (A2) and (A5) expressed in terms of Ω(T), a measure of temperature sensitivity, as evaluated via Equation (7). It is noted that curve 1 is based upon the fit obtained in Appendix B based (mainly) upon recoverable shear creep data between 100 and 135 °C. On the other hand, curves 2 and 3 should both be applicable up to 300 °C, based upon the related results in Figure 3 and Figure A1, respectively. It is noted that curve 2 lies consistently above curve 1, that is, with increasing temperature, $\tau_{\mathrm{seg},c}$ in Equation (12) consistently decays faster (on a fractional basis) than $a_{T}$ in Equation (A5). On the other hand, it is noted from Figure 4 that curve 3, corresponding to $a_{T}$ for the flow regime in Appendix A, intersects curves 1 and 2 at the respective temperatures of 115.9 °C (as can also be seen from Figure 1 above) and 158.5 °C.

Making use of the three curves in Figure 4, one might address some related results in the literature. For example, Figure 3 of He et al. [5] seems to convincingly indicate that the segmental correlation time and viscosity have the same temperature dependence. In particular, the most extensive viscosity results in their Figure 3 are for the “PS-2” polymer, in terms of eight (solid square) data points that range between 1000/T ≅ 2.046/°K on the left and 2.493/°K on the right. Adjusting these points by ΔT_g_ (=373 °K–331 °K = 42 °K, according to their Figure 9 and Table 1) to account for a small M_w_ of 2310, we are then dealing (in terms of °C) with the temperatures of about 257 °C and 170 °C, respectively. Based on Equations (A1) and (A2) of Appendix A, it follows that curve 3 in Figure 4 corresponds to

(13) $$\frac{a_{170\;{}^{\circ}C}}{a_{257\;{}^{\circ}C}}=2.960\;\times \;10^{2}$$

whereas, from Equation (12), curve 2 in Figure 4 corresponds to

(14) $$\frac{\tau_{\mathrm{seg},c\;(170\;{}^{\circ}C)}}{\tau_{\mathrm{seg},c\;(257\;{}^{\circ}C)}}=1.678\;\times \;10^{2}$$

In particular,

2.960 × 10^2^/1.678 × 10^2^ = 1.764 = 10^0.246^ (15)

That is, according to the tight correlation for the viscosity of PS given by Equations (A1) and (A2), the result in Equation (15) indicates that the total variation in a_T_ between the left-most and the right-most solid squares in Figure 3 of He et al. [5] exceeds that of τ_seg,c_(T), given in Equation (12), by a factor of 1.764. On a logarithmic basis, as in their Figure 3, this becomes a difference of 0.246 decades. {Note: if a_T_ in Equations (A1) and (A2) is replaced by Equations (A1) and (A3), namely, the fit obtained by McKenna et al. [28], the difference becomes 0.263 decades.} Accordingly, such results call into question the close correlation between viscosity and τ_seg,c_ for the polymer d_8_PS-2 shown in Figure 3 of [5].

Another concern relates to the relative behavior of creep compliance and viscosity at higher temperatures. In this regard, one might refer to the work of Ngai [29,30], namely, pp. 117/118 from [29] and pp. 262/263 from [30]. In both cases, Ngai first stated that, above ≈407 °K (134 °C), the (recoverable) creep compliance and viscosity have the same a_T_. However, Ngai then qualified this by indicating that extrapolating creep compliance data to higher temperatures indicates a weaker temperature dependence than for viscosity. Unlike in [29], Ngai’s corresponding plot in [30], namely, Figure 101 (left panel, for PS), explicitly includes a curve for (recoverable) creep compliance that decays more slowly than that for viscosity. In turn, this is in agreement with the present results, as shown in Figure 4, where curve 1 (corresponding to recoverable creep compliance) lies below curve 3 (viscous flow) at higher temperatures, namely, above 115.9 °C. Since Ω characterizes the temperature sensitivity, the lower value for curve 1 indicates a slower decay with increasing temperature.

It is noted that Ngai [30] indicated (on p. 263) that the segmental relaxation time (τ_α_) has the same temperature dependence as creep compliance up to 384 °K (111 °C); indeed, this agrees with the present results shown in Figure 2, in which there is excellent agreement with the curve, based upon Equations (8), (9) and (11), up to about 111 °C. On the other hand, there is also evidence that Equations (8), (9) and (11) describe τ_α_ (T) even below T_g_. This is based upon results from Hintermeyer et al. [23], as documented in the following section.

## 6. Unanticipated Corroboration

In Figure 11 of Hintermeyer et al. [23], based on dielectric spectra data for PS, the results are plotted in terms of “lg τ_α_ (s)” versus “z ≡ m (T/T_g_ − 1)”, in which “m” is the non-dimensional “fragility index”. Of specific interest here is the fact that the data (all of which lie essentially above T_g_) coalesce asymptotically onto a straight line as one approaches T_g_ (identified with τ_α_ ≡ 10^2^ s) from above. In particular, the straight line in their Figure 11 corresponds to

(16) $$\log\;{\tau_{\alpha }}_{\;}=2-\frac{m}{T_{g}}(T\;-\;T_{g})$$

where τ_α_ is in seconds, and T and T_g_ in °K. From their Figure 6, m ≈ 122 for the three samples with the largest M_w_ and T_g_ ≅ 373°K. Hence, Equation (16) becomes

τ_α_ (T) = 10^2^ s exp{−(0.75/°C) (T − T_g_)} (17)

for the PS polymers of high M_w_. Indeed, the value of Ω = 0.75/°C in Equation (17) agrees well (within 7%) with the value of 0.805/°C in Equation (5). This is evidenced by the dashed line in Figure 2, which is based upon Equation (17) with T_g_ = 100 °C.

Hence, there is a strong implication that the present correlation, corresponding to Equations (8), (9) and (11) and plotted as the curve in Figure 2 above, describes τ_α_ (T) for PS not only up to 111 °C but also down to, perhaps, 83 °C, based on the density relaxation results for PS presented by Hieber [1], upon which the value of 0.805/°C is based.

In a similar manner, based upon the DS data of Hintermeyer et al. [23] for polydimethylsiloxane (PDMS) and polybutadiene (PB), one obtains the respective values for Ω of 1.83/°C and 1.19/°C, based upon the higher M_w_ samples. However, since this relates to the segmental relaxation time, the same values for Ω also pertain (essentially) to the polymers of lower M_w_.

In particular, based upon Equations (3) {with “τ_eq_” replaced by “τ_α_”} and (16), it follows that

(18) $$\Omega =\ln\;10\times \frac{m}{T_{g}\left({}^{\circ}K\right)}$$

where Ω (1/°K) or, equivalently, Ω (1/°C). Hence, based on the values for m (fragility index) presented by Hintermeyer et al. [23] in their Figures 5c, 6c and 7c for PDMS, PS, and PB, respectively, with corresponding values for T_g_ (°K) from their Tables 1–3, the resulting values are presented in Table 1, Table 2 and Table 3 below for Ω (1/°C) as a function of M_w_.

## 7. Conclusions

The main results that were obtained in the present paper include the following:

(i) It was shown, making use of the extensive DS data of Hintermeyer et al. [23] for PS {as well as for polydimethylsiloxane (PDMS) and 1, 4-polybutadiene (PB)}, that the temperature dependence of the local segmental relaxation time, τ_α_, is purely exponential below T_g_, thus confirming previous results for PS obtained by Hieber [1], based on density relaxation considerations.
(ii) The fact that the values of 0.805/°C in Equation (5) and 0.75/°C in Equation (17) are in such close agreement strongly suggests that τ_eq_ (T), obtained from density relaxation considerations, and τ_α_ (T), obtained from segmental relaxation considerations, are directly related.
(iii) The results shown in Figure 2 indicate that the smooth composite correlation (with no adjustable parameters) given by Equations (9) and (11) describes the available experimental results for the segmental relaxation time of PS encompassing a definite temperature range both above and below the glass transition temperature.
(iv) Based upon the results in Section 5, there is strong evidence that, contrary to some results in the literature, the temperature dependence of τ_α_(T), given in Equation (12), and of $a_{T}$ for viscosity, given in Equations (A1) and (A2), does not become coincident at higher temperatures.

## Figures and Tables

**Table 1:** Ω (1/°C) results for PDMS, based on Equation (18) and Table 1 and Figure 5c of Hintermeyer et al. [23].

M_w_	T_g_ (°K)	m	Ω (1/°C)
311	126.3	107.1	1.95
311	126.3	116.1	2.12
860	133.6	100.9	1.74
1600	138.2	108.0	1.80
1600	138.2	110.0	1.83
2490	139.9	110.0	1.81
3510	141.5	111.0	1.81
4560	142.4	111.4	1.80
5940	143.0	113.2	1.82
11.0 K	144.0	115.1	1.84
21.6 K	144.2	113.6	1.81
41.4 K	144.5	114.1	1.82
128 K	144.4	116.1	1.85
232 K	144.6	115.0	1.83

**Table 2:** Ω (1/°C) results for PS based upon Equation (18) and Table 2 and Figure 6c of Hintermeyer et al. [23].

M_w_	T_g_ (°K)	m	Ω (1/°C)
106	114.7	81.6	1.64
162	138.9	76.8	1.27
370	231.9	78.0	0.77
690	261.4	72.1	0.64
1350	314.2	97.0	0.71
3250	347.0	107.5	0.71
8900	356.9	105.4	0.68
19.1 K	367.9	116.2	0.73
33.5 K	369.0	119.6	0.75
96.0 K	372.6	120.9	0.75
243 K	373.3	122.6	0.76
546 K	372.0	122.0	0.76

**Table 3:** Ω (1/°C) results for PB based upon Equation (18) and Table 3 and Figure 7c of Hintermeyer et al. [23].

M_w_	T_g_ (°K)	m	Ω (1/°C)
355	140.9	70.0	1.14
466	161.2	71.1	1.02
575	162.1	73.0	1.04
777	165.3	78.0	1.09
1450	170.7	81.1	1.09
2020	173.6	83.0	1.10
2760	174.5	88.0	1.16
4600	174.0	90.9	1.20
19.9 K	175.3	90.0	1.18
35.3 K	174.5	90.9	1.20
87.0 K	174.4	90.5	1.19

## Data Availability

The original contributions presented in the study are included in the article; further inquiries can be directed to the corresponding author.

## Appendix A. $a_{T}$ for PS in “Flow Regime”

Shown in Figure A1 are the results for the temperature shift factor $a_{T}$/a_160°C_ for the “flow regime” of PS based on available data. In particular, the results from Plazek [9] are based on (long-time) shear creep compliance, whereas the remaining data are all based upon dynamic measurements in the low-frequency limit. The results from Zosel [10] are significant in that they include some eleven different PS samples (all with M_ν_ > 150 K, of both narrow and broad molecular weight distribution), for which $a_{T}$ was essentially the same. In a similar manner, Marin and Graessley [11] found that, within experimental uncertainties, their results for $a_{T}$ were the same for their five PS samples (all of NMWD, with M_w_ between 37 K and 670 K). Similarly, Schausberger et al. [12] found that, for five PS of NMWD (with M_w_ ranging between 70 K and 3000 K), $a_{T}$ was the same within the accuracy of the measurements. In turn, the experimental results from these three investigations were complemented in Figure A1 by the results of Plazek [9] and Lomellini [13], extending the results to lower and higher temperatures, respectively.

Also shown in Figure A1 is the corresponding curve fit based upon the VFTH model [6,7,8]:

(A1) $$a_{T}/a_{\mathrm{TREF}}=\exp\left(\frac{\mathrm{CC}}{{T-T}_{\infty }}\right)/\exp\left(\frac{\mathrm{CC}}{{\mathrm{TREF}-T}_{\infty }}\right)$$

with TREF = 160 °C. The best-fit values for (CC, T_∞_) were determined by employing a simplex method (Nelder and Mead [31]), resulting in

CC = 1793.8 °C, T_∞_ = 42.27 °C, RMSDEV = 0.03994. (A2)

For comparison, it should be noted that McKenna et al. [28] performed a similar analysis of mostly different data for PS. The only viscosity measurements common to the earlier 1987 paper and the present investigation are those of Plazek [9] and Marin and Graessley [11]. Based upon fitting an extensive set of measurements, the resulting best-fit values based upon the VFTH model were found by McKenna et al. [28] to be given by

CC = 1794 °C, T_∞_ = 42.8 °C. (A3)

The remarkably close agreement between the model parameters in Equations (A2) and (A3) thus serves as a confirmation of the two separate investigations.

## Appendix B. $a_{T}$ for PS in “Glass–Rubber Transition”

Shown in Figure A2 are the results for the temperature shift factor, $a_{T}$/$a_{115{}^{\circ}C}$, for the “glass–rubber transition” regime of PS based upon the available data. In particular, the results of Aklonis and Tobolsky [14] are based upon stress-relaxation measurements, whereas the remaining data are all based upon recoverable-shear-creep results.

The two curves in Figure A2 are both based upon the VFTH model, as given in Equation (A1), now with TREF = 115 °C, with the dashed curve being the best fit of all indicated data, with

CC = 669.8 °C, T_∞_ = 71.60 °C, RMSDEV = 0.1869 (A4)

and the solid curve being the best fit when the data of Plazek and O’Rourke [15] are omitted, resulting in

CC = 714.4 °C, T_∞_ = 69.43 °C, RMSDEV = 0.09965. (A5)

It is noted that the RMSDEV is almost halved by omitting the data of Plazek and O’Rourke [15], indicating that the latter data are anomalous.

Although Plazek and O’Rourke [15] presented tabulated results for nine PS samples of NMWD, only the four samples with the highest molecular weight (94 K, 189 K, 600 K, and 800 K) were included in Figure A2. (A fifth polymer, of molecular weight 47 K, was actually from Plazek [9] and was plotted in Figure A2 accordingly.) It is noted that the shift factor for recoverable compliance in Table II of Plazek and O’Rourke [15] is relative to 100 °C. However, when expressed relative to 115 °C (which was chosen as a representative value, given the temperature range of the data in Figure A2), the results from Plazek and O’Rourke [15] become clearly anomalous in the lower-temperature range, because those data decay much more rapidly between 100 and 115 °C (as can be seen from Figure A2) than the remaining experimental results from the other five sources.
